# Improving Rice Root Development and Soil Health in Saline Soils: A Biochar and Microbial-Inoculated Biochar with Nitrogen Approach

**DOI:** 10.3390/plants15060986

**Published:** 2026-03-23

**Authors:** Hafiz Muhammad Mazhar Abbas, Song Li, Wentao Zhou, Haider Sultan, Mohammad Nauman Khan, Asad Shah, Ashar Tahir, Hamza Iltaf, Yixue Mu, Lixiao Nie

**Affiliations:** 1 Sanya National Center of Technology Innovation for Saline-Alkali Tolerant Rice, School of Breeding and Multiplication (Sanya Institute of Breeding and Multiplication), Hainan University, Sanya 572025, China; mazharabbas@hainanu.edu.cn (H.M.M.A.); 23210901000080@hainanu.edu.cn (S.L.); 23220951310200@hainanu.edu.cn (W.Z.); 184268@hainanu.edu.cn (M.N.K.); 184466@hainanu.edu.cn (A.S.);; 2 Hubei Engineering Research Center for Protection and Utilization of Special Biological Resources in the Hajiang River Basin, College of Life Sciences, Jianghan University, Wuhan 430056, China; sultanhaider@hainanu.edu.cn; 3 School of Tropical Agriculture and Forestry, Hainan University, Haikou 570228, China; drashartahir@hainanu.edu.cn; 4 School of Breeding and Multiplication (Sanya Institute of Breeding and Multiplication), Sanya 572000, China

**Keywords:** saline water, root morphology, microbial biochar, simple biochar

## Abstract

This study investigated the combined effects of microbial-inoculated biochar and nitrogen (N) on rice growth and soil properties under saline conditions. A randomized complete block design with three replications was employed to evaluate three factors: (i) salinity level (non-saline, S0; saline, 0.4% NaCl, S1), (ii) biochar type (20 t/ha BC, BF, BB, and BFB), and (iii) nitrogen application rate (60 and 120 kg ha^−1^). Soil physicochemical and biological properties, along with rice root development, were assessed. Salinity significantly reduced soil organic matter (OM) by 9%, nitrate nitrogen (NO_3_^−^-N) by 16%, ammonium nitrogen (NH_4_^+^-N) by 8.18%, and available phosphorus (AP) by 6.81%. Soil enzyme activities, including catalase (CAT), acid phosphatase (ACP), polyphenol oxidase (POX), and β-D-glucosidase (BG), decreased by 32.69%, 29%, 39.18%, and 19.44%, respectively, resulting in suppressed root growth compared with non-saline conditions. The combined treatment of microbial biochar (BFB) and N at 120 kg ha^−1^ (BFB + N120) markedly improved saline soil quality and rice root performance by maintaining a favorable K^+^/Na^+^ balance in roots. Specifically, BFB+N120 increased OM by 145% and 120% compared with N120 and BC alone, respectively, and enhanced NO_3_^−^-N, NH_4_^+^-N, and soil enzyme activities (CAT, ACP, POX, and BG). These improvements were strongly associated with enhanced root development. Under saline conditions, BFB+N120 increased root dry mass by 429% and 1185.71%, and root length by 63% and 83%, compared with N120 and BC alone, respectively, in the cultivar Jing Liang You 534. Overall, the results demonstrate that microbial-modified biochar combined with nitrogen fertilizer mitigates salt-induced soil degradation by improving physicochemical and biological properties, thereby enhancing nutrient availability, ionic homeostasis, and root growth. This study provides mechanistic insights into the combined role of microbial biochar and nitrogen in the remediation of saline soils.

## 1. Introduction

Soil salinization and the use of saline irrigation water are among the most critical abiotic stresses threatening global food security by reducing crop productivity and degrading soil health [1,2,3], with freshwater resources becoming increasingly scarce worldwide [4]. The use of saline irrigation water especially without an appropriate leaching fraction leads to accumulation of salt especially sodium salts [5]. However, Wang et al. [6] reported that the higher Na^+^ concentration has a direct toxicity effect, but also adversely affects soil structure. This in turn leads to a nutrient imbalance, lower microbial health and hence lower productivity. Irrigating with saline water enhances soil salinization, leading to a significant decline in agricultural soil quality and causing physicochemical deterioration [6], due to salt accumulation in the rhizosphere [7].

Plant roots are the first plant organs to come in contact with saline-affected soil [8]. Many studies have reported that salt stress has negative effects on root growth [9]. In addition to deteriorating soil physicochemical properties, salinity directly affects plant growth and metabolism by lowering soil water potential, which induces physiological drought and restricts water uptake by roots. Excessive accumulation of Na^+^ and Cl^−^ ions disrupts ionic homeostasis and competes with essential nutrients, such as K^+^ and Ca^2+^, leading to a nutrient imbalance [2]. These ionic and osmotic stresses impair photosynthesis, enzyme activity, and cellular metabolism, ultimately reducing root and shoot biomass and altering the chemical composition of plant tissues, including carbon, nitrogen, and mineral nutrient contents [2]. Among soil amendments, biochar has emerged as a novel soil amendment for improving the properties of salt-affected soils [10,11]. In their study, Tejada et al. [12] suggested some ways to deal with salty irrigation water, like choosing salt-tolerant plants, using smart watering techniques, improving planting methods, and using special soil treatments to make the soil healthier. Moreover, Cui et al. [13] and Yin et al. [14] reported that the combined effect of biochar and microorganisms offers a novel and sustainable approach to enhance plant growth and remediate both saline and alkaline soils. Additionally, the interaction between microbes and biochar has been shown to enhance both belowground and aboveground plant biomass by improving soil properties and nutrient availability [15,16,17]. Moreover, nitrogen (N) application alongside biochar has been shown to further improve soil quality by increasing nitrogen use efficiency (NUE), reducing N losses, and enhancing microbial function [18]. Similarly, it was also reported that the combined application of biochar and N enhanced the efficiency of biochar in mitigating negative effects of abiotic stresses as N has positive effects on soil biological properties [19]. Biochar mitigates soil salinity by improving soil structure and water-holding capacity, adsorbing excess Na^+^ and Cl^−^ ions, and increasing cation exchange capacity, which enhances the availability of essential nutrients and reduces ionic imbalance. Additionally, biochar promotes soil aggregation and microbial activity, facilitating salt leaching from the root zone and improving rhizosphere conditions. Collectively, these mechanisms reduce osmotic and ionic stress on plants and enhance their tolerance to saline environments [15,18]. A cbined combined application of biochar and microorganisms enhanced soil fertility and plant productivity by improving nutrient retention and availability through the porous structure of biochar, while microorganisms further facilitated nutrient cycling and plant growth promotion, leading to combined effects that surpassed the benefits of either amendment applied alone [2]. Additionally, Ali et al. [20] reported that the combined effect of biochar and lower N application rates improved soil properties and ultimately plant growth and yield. In the recent decade, few studies have addressed how combined microbial inoculation (bacteria and fungi) on biochar, along with nitrogen fertilizer application, influences salt-mitigating microbial communities, soil physicochemical properties, and root morphology in saline-irrigated paddy fields. After collection, the raw material (rice residues) was cleaned to remove surface impurities and then air-dried. The dried biomass was conveyed via a conveyor belt to a crusher, where it was mechanically ground to a uniform particle size. The crushed material was subsequently fed into a charcoal furnace through the feeding system. Pyrolysis was initiated by gradually increasing the temperature under controlled conditions, and the furnace temperature was maintained at 500 °C. Carbonization was carried out under low-oxygen, high-temperature conditions for approximately 2–3 h. After the completion of carbonization, the biochar was cooled to approximately 25 °C using a cooling system, and the material was discharged intermittently through the feeding pipe to obtain the final biochar product.

This knowledge gap limits our understanding of how such integrated strategies can enhance plant resilience and soil health in salt-affected environments.

Based on the above-mentioned investigations of using biochar, microorganisms and nitrogen, we proposed following hypothesis.

(1) Combined inoculation of salt-tolerant bacteria and fungi onto biochar can create a favorable microbial environment that enhances salt stress tolerance in rice.
(2) Nitrogen fertilizer application further strengthens the effect of microbial-inoculated biochar by enhancing microbial activity and improving soil quality (soil organic matter, nitrate and ammonium nitrogen, available phosphorus, and soil enzyme activities CAT, ACP, POX, and β-D-glucosidase), thereby promoting root development under saline conditions.

## 2. Results

### 2.1. Characterization of Biochar

**Figure 1:**
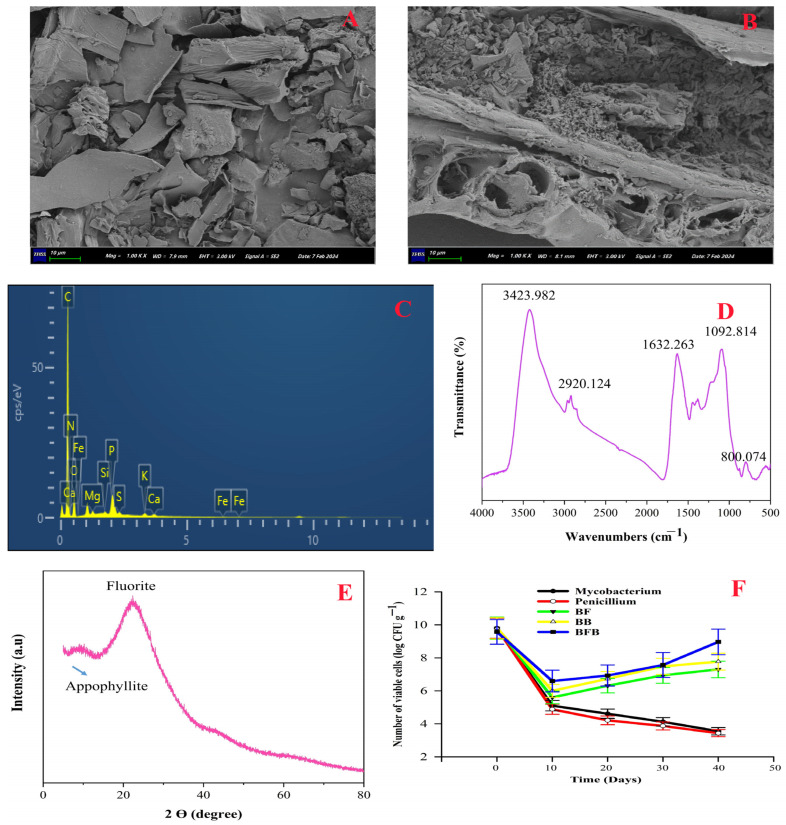
Characterization of biochar; SEM analysis of BC (**A**), SEM analysis of BFB (**B**), EDS analysis of BFB (**C**), FTIR of BFB (**D**), XRD of BFB (**E**), and number of viable bacterial (Mycobacterium-black) and fungal (Penicillium-red) cells, along with microbial-inoculated biochar treatments, bacteria-loaded biochar (BB-yellow), Penicillium-inoculated biochar (BF-green) in salt-contaminated soil and bacterial–fungal-inoculated biochar (BFB-blue) expressed as log_10_ CFU g^−1^ (mean ± SD, n = 3) (**F**).

Rice husk biochar prepared at 500 °C showed moderate carbon stability, reflected by an H/C atomic ratio of 0.95, low moisture content (5%), and a powdered granular structure, indicating its potential for sustained persistence and functional performance in soil systems. The cation exchange capacity (CEC) of the biochar used in this study was 140 cmol(+) kg^−1^, which contributes to improved nutrient retention and ion exchange in saline soils. However, this value is lower than that of natural humic substances. According to Stevenson [21], the CEC of humus typically ranges from 300 to 1400 cmol kg^−1^, indicating that humic substances possess a greater density of functional groups responsible for cation exchange. Nevertheless, biochar can still significantly enhance soil CEC and nutrient retention due to its porous structure and surface functional groups formed during pyrolysis. Scanning electron microscopic (SEM) analysis of BFB revealed a porous biochar surface densely populated with microbes. This structure may facilitate microbial adhesion and growth on the biochar, as observed by numerous bacterial and fungal cells either dispersed or clustered on the biochar surface (Figure 1A). Due to their small size (<2 µm), it is possible that some microbial cells entered the biochar pores. The successful colonization of biochar by bacteria and fungi is primarily determined by their physiological characteristics and the physicochemical attributes of the biochar [22]. High surface area, porous structure and mechanical stability of biochar facilitate the electrostatic interactions, hydrogen bonding, and hydrophobic interactions between microorganisms, and the biochar surface makes it an ideal substrate for microbial growth and microbial adhesion [23,24,25]. It was also reported that SEM (scanning electron microscopy) analysis demonstrated that biochar, with its high specific surface area, laminar structure, and abundant pores, provides an optimal environment for microbial attachment and stimulates extracellular polymeric substance (EPS) production, as evidenced by the dense colonization, interwoven mycelia, and nonlinear biofilm networks observed on its surface, in contrast to the loosely structured flocs without biochar [16,26]. These observations suggest that biochar can effectively stimulate microbial EPS production. Similarly, Bolan et al. [27] reported that the modifications of biochar can ultimately alter the surface properties, morphology, composition, and number of oxygen-containing functional groups, increase the surface area of adsorption sites, and enhance microbial attachment to the biochar surface. Furthermore, biochar’s surface harbors functional groups (hydroxyl, carboxyl, phosphate, amine, and sulfhydryl) capable of interacting with analogous groups present on bacteria and fungi [28]. These interactions, including ionic bonding, hydrogen bonding, and van der Waals forces, promote microbial adhesion to the biochar. Elemental (EDS) analysis showed that carbon and oxygen were the predominant elements in all biochar types. In BFB, sodium was present at a relatively low concentration, whereas potassium was detected at a higher concentration of 0.45% (Figure 1B).

To elucidate the interactions between biochar’s functional groups and fungi and bacteria, FTIR spectroscopy was employed (Figure 1C). The prominent spectral peak observed at approximately 3423.982 cm^−1^ corresponds to the stretching vibrations of hydroxyl (–OH) and amino (–NH) functional groups, which are commonly reported surface functional groups in biochar [29]. The spectral peak located at 2920.124 cm^−1^ is assigned to the stretching vibrations of carbon–hydrogen (C-H) bonds within alkyl groups, which are structural components of the biochar [30]. The broad peak centered around 1632.263 cm^−1^ suggests bending vibrations associated with amide I and amide II bonds. These bonds are prevalent in proteins and other molecules containing peptide groups [31].

The X-ray diffraction patterns obtained from BFB are illustrated in Figure 1D. The X-ray diffraction patterns of BFB exhibit a broad peak within the 15–30-degree 2θ range. This peak is attributed to amorphous carbon reflections, indicating a disordered structure composed of randomly oriented aromatic carbon sheets within all biochar samples. XRD analysis of bacterial–fungal-loaded biochar revealed a narrower peak within the 2θ range of 10–30°, indicative of the presence of diverse mineral crystals and other inorganic materials (Figure 1D). The X-ray diffraction pattern exhibited additional minor peaks, and due to their overlapping nature, a qualitative analysis approach was utilized. Bacterial–fungal biochar exhibited a distinct peak at approximately 28° (2θ), which likely corresponds to crystalline mineral phases derived from inherent biomass ash components (e.g., silica), rather than indicating the definitive presence of fluorite. Moreover, Novak et al. [32] reported the hydroxylation of aromatic biochar compounds by Mycobacterium sp. Moreover, Zimmerman [33] observed higher mineralization rates, approximating 20 mg C g^−1^ char, in microorganism-inoculated compared to sterilized incubations.

### 2.2. Counts of Free and Biochar-Inoculated Microbes Under Saline Conditions

Experimental results showed a gradual decline in the population of free-living microorganisms throughout the incubation period. In contrast, microbial populations immobilized on biochar (BF, BB, and BFB) exhibited an initial decrease in viable cell counts during the first 10 days, followed by stabilization and sustained survival over time (Figure 1F). Overall, biochar-immobilized microorganisms demonstrated markedly higher survival under saline soil conditions than free-living cells. Among the treatments, viable cell densities under saline conditions followed the order BFB > BB > BF > free Mycobacterium spp. > free Penicillium spp., indicating the superior protective effect of bacterial–fungal biochar immobilization.

### 2.3. Chemical Properties of Post-Harvested Soil

**Figure 2:**
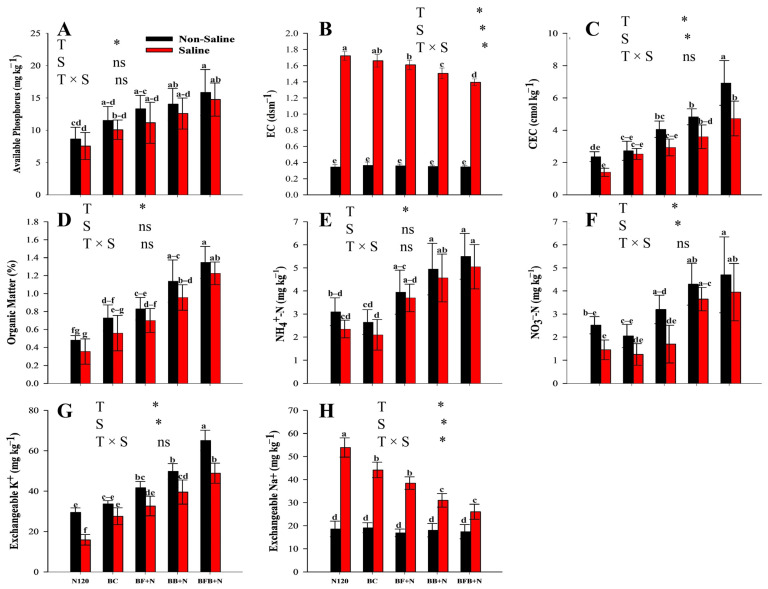
Effect of microbial-inoculated biochar and N fertilizer on chemical properties of soil; AP (**A**), EC of soil (**B**), CEC of soil (**C**), OM of soil (**D**), NH_4_^+^-N of soil (**E**), NO_3_^−^-N of soil (**F**), exchangeable K^+^ contents of soil (**G**), exchangeable Na^+^ of soil (**H**). The means that have the same letter do not differ substantially at *p* > 0.05 for a parameter. Significant * (*p* ≤ 0.05); non-significant (ns; *p* > 0.05). T (treatment); S (salt).

Statistical analysis clearly demonstrated that salinity exerted adverse effects on key soil properties (Figure 2). Significant N × biochar and biochar × salinity interactions (Table 1) indicate a combined effect of microbial-inoculated biochar and nitrogen in mitigating salinity stress. Under saline conditions, significant differences were observed among treatments in available phosphorus (P), electrical conductivity (EC), cation exchange capacity (CEC), organic matter (OM), ammonium nitrogen (NH_4_^+^-N), nitrate nitrogen (NO_3_^−^-N), exchangeable potassium (K^+^), and exchangeable sodium (Na^+^) versus non-saline conditions. Overall, treatments combining bacterial–fungal biochar with nitrogen (BFB+N) consistently outperformed nitrogen (N120) or biochar (BC) applied alone, followed by the BB+N treatment.

Specifically, the BFB+N120 treatment increased soil-available P by 7% under saline compared with non-saline conditions (Figure 2A), while simultaneously reducing soil EC by 19% and 12.6% relative to the same controls (Figure 2B). Soil CEC was markedly enhanced under BFB+N120, showing increases of 238% and 31.5% compared with N120 and BC treatments, respectively (Figure 2C). Similarly, soil organic matter content was substantially higher under BFB+N120, increasing by 120% relative to BC alone.

The interaction between BFB and N120 also significantly enhanced inorganic nitrogen availability, with NH_4_^+^-N and NO_3_^−^-N increasing by 100% compared with N120 and by 115–172% compared with BC treatments (Figure 2E,F). Exchangeable K^+^ content was significantly higher under BFB+N120, increasing by 207.4% and 77.3% relative to N120 and BC treatments, respectively. Although salinity increased soil Na^+^ levels across treatments, the BFB+N120 treatment effectively reduced exchangeable Na^+^ by 51.6% and 41% compared with N120 and BC alone (Figure 2H).

**Figure 3:**
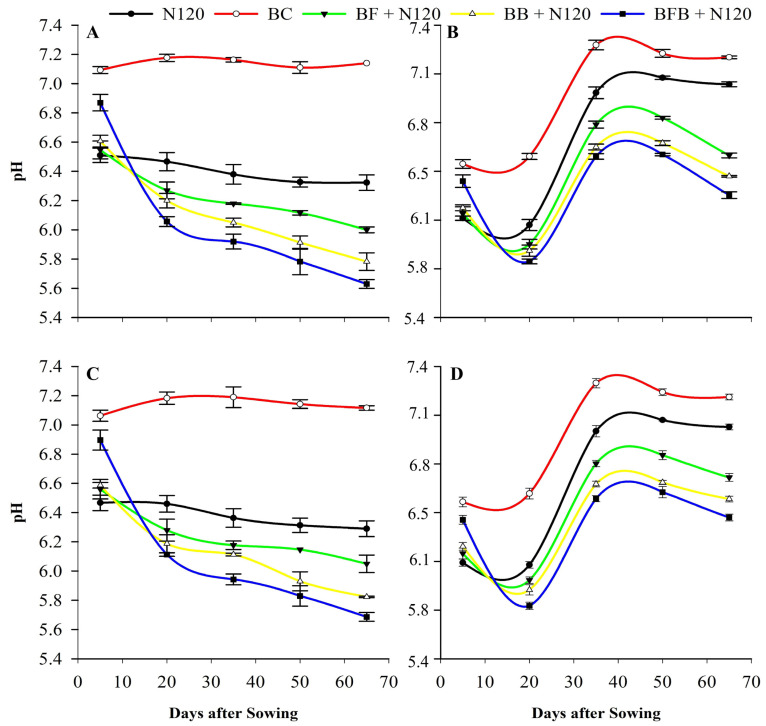
Effects of different treatments on soil pH during experiment; soil pH of non-saline conditions for SLY138 (**A**), soil pH of saline conditions for SLY138 (**B**), soil pH of non-saline conditions for JLY534 (**C**), and soil pH of saline conditions for JLY534 (**D**). Values are the mean ± SD; error bars represent the standard error of the three repeats.

Soil pH dynamics varied among treatments throughout the incubation period (Figure 3). Interaction terms were not significant for most variables (Table 1); therefore, results are presented and discussed based on main effects. All biochar-based treatments, including microbial-loaded biochars and BC alone, increased soil pH during the early incubation stage (day 5), with BC showing the strongest alkalizing effect. Over time, soil pH generally declined across treatments; however, BC exhibited an increasing trend throughout the incubation period. A transient rise in soil pH was observed between days 20 and 35 following saline water application. Thereafter, BF+N, BB+N, and BFB+N treatments showed a more pronounced decrease in soil pH compared with their respective single BC and N treatments, continuing until harvest (day 65).

### 2.4. Microbial Community Composition in Saline Soil

**Figure 4:**
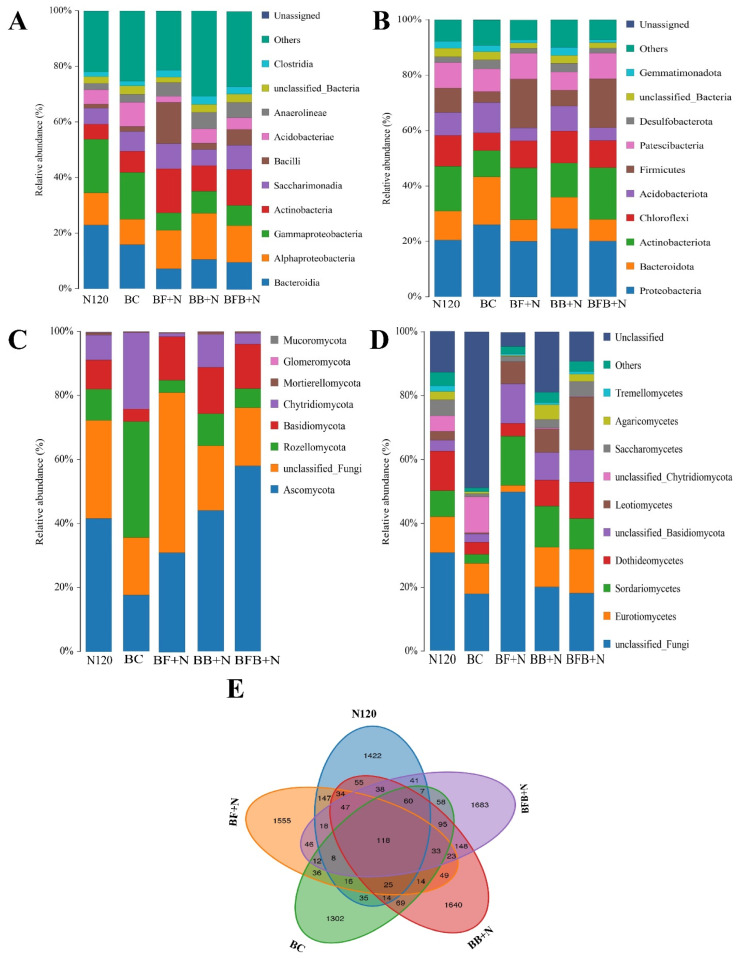
Effects of the nitrogen (N120), simple biochar (BC), and combined effect of fungal, bacterial and bacterial–fungal biochar with nitrogen (BF+N, BB+N and BFB+N) on the relative abundance of bacterial phylum (**A**), bacterial species (**B**), fungal phylum (**C**), fungal species (**D**), and relative abundance of bacterial species in different treatments (**E**) in saline soil.

Microbial community composition was comprehensively analyzed across all treatments under saline conditions (Figure 4). At the bacterial level, relative abundance followed the order BC > N120 > BF+N > BB+N > BFB+N (Figure 4E). Across all treatments, the dominant bacterial phyla were Proteobacteria, Bacteroidota, Chloroflexi, Acidobacteriota, and Actinobacteriota, collectively accounting for more than 70% of the total bacterial community under saline conditions. Among these, Proteobacteria contributed over 20% of the total bacterial diversity and showed a marked variation among treatments, which corresponded closely with differences in soil Na^+^ content (Figure 4B). In contrast, Chloroflexi and Actinobacteriota were relatively more abundant in the BF+N, BB+N, and BFB+N treatments, whereas Proteobacteria, Acidobacteriota, and Bacteroidota were more prevalent in the N120 and BC treatments.

At the class level, the bacterial community was dominated by Gammaproteobacteria, Acidobacteria, Deltaproteobacteria, Anaerolineae, Bacteroidia, Flavobacteria, and Alphaproteobacteria (Figure 4B). Gammaproteobacteria was the most abundant class in saline soils treated with N120 and BC, while Anaerolineae and Bacilli were detected at higher relative abundances in the BF+N, BB+N, and BFB+N treatments, indicating a distinct shift in bacterial community structure following microbial-loaded biochar application.

Fungal community analysis revealed that Ascomycota and Basidiomycota were the dominant phyla in soils treated with N120, BF+N, BB+N, and BFB+N under saline conditions (Figure 4C). In contrast, Rozellomycota and Chytridiomycota were relatively more abundant in the BC treatment. At the class level, Eurotiomycetes, Sordariomycetes, Dothideomycetes, and Leotiomycetes consistently dominated fungal communities across all treatments (Figure 4D), suggesting that salinity exerted a strong selective pressure on fungal community composition regardless of treatment.

**Figure 5:**
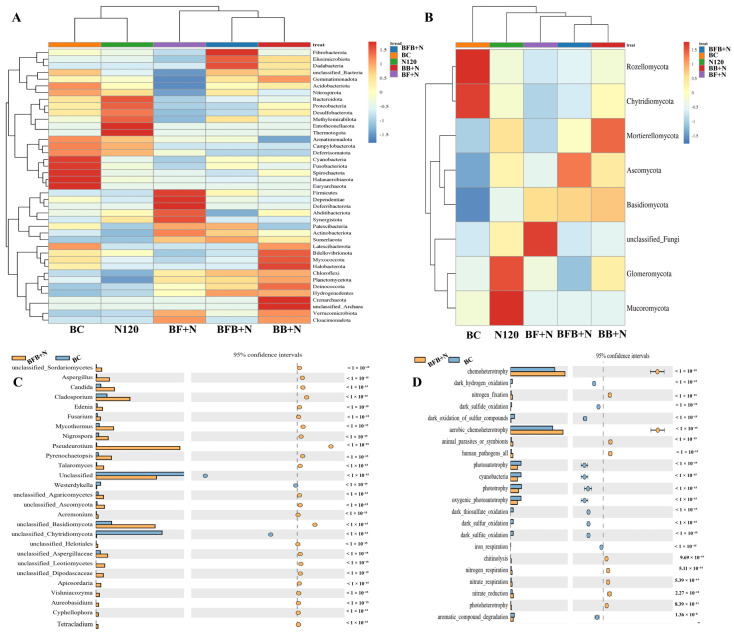
Differential abundance of bacteria at phylum level (**A**), fungi at phylum level (**B**), extended error bar plot of fungal phylum between BC and BFB+N (**C**), and N-related functions by bacteria in different treatments (**D**).

To further identify treatment-specific microbial responses, mean species abundance was compared among treatments (Figure 5). Bacterial taxa such as Gemmatimonadota and fungal groups including Ascomycota and Basidiomycota, which are closely associated with organic matter decomposition, were markedly enriched in the BFB+N treatment (Figure 5A,B). Under saline conditions, the BFB+N treatment showed superior performance by promoting the abundance of Dadabacteria while suppressing Campilobacterota, indicating a selective enrichment of salt-tolerant and functionally beneficial microbial groups (Figure 5A). In addition, microbial functions related to nitrogen cycling—including nitrogen respiration, nitrogen fixation, and nitrogen reduction—were most pronounced in the BFB+N treatment. Consistently, fungi involved in nitrogen and carbon recycling, particularly Aspergillus, were also most abundant under BFB+N application (Figure 5C,D).

### 2.5. Effect of Microbial Biochar on Enzyme Activities in Saline Soil

**Figure 6:**
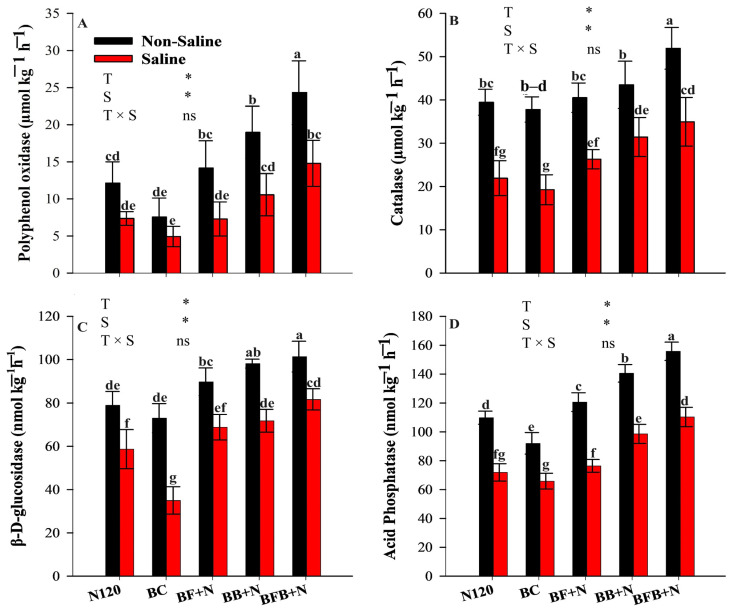
Effect of microbial biochar and N on enzyme activities of soil; POX (**A**), CAT (**B**), BG (**C**), and AP (**D**). The means that have the same letter do not differ substantially at *p* > 0.05 for a parameter. Significant * (*p* ≤ 0.05); non-significant (ns; *p* > 0.05). T (treatment); S (salt).

Soil enzymatic activities differed significantly among treatments under both saline and non-saline conditions (*p* < 0.05; Figure 6). Under saline stress, the BFB+N treatment significantly enhanced the activities of β-D-glucosidase (BG), acid phosphatase (AP), polyphenol oxidase (POX), and catalase (CAT) by 39.2–133.5%, 53.5–67.5%, 100.7–200.5%, and 59.4–81.5%, respectively, compared with N120 and BC treatments. Similar trends were observed under non-saline conditions, where BG, AP, POX, and CAT activities in the BFB+N treatment were 28.5–38.5%, 41.9–69.3%, 28.2–100%, and 31.6–37.4% higher, respectively, than those in N120 and BC treatments. These results collectively demonstrate that microbial-inoculated biochar combined with nitrogen effectively stimulates soil microbial functionality and enzymatic activity across contrasting salinity conditions.

### 2.6. K^+^/Na^+^, Morphological and Anatomical Attributes of Roots in Rice Roots Under Saline Conditions

**Figure 7:**
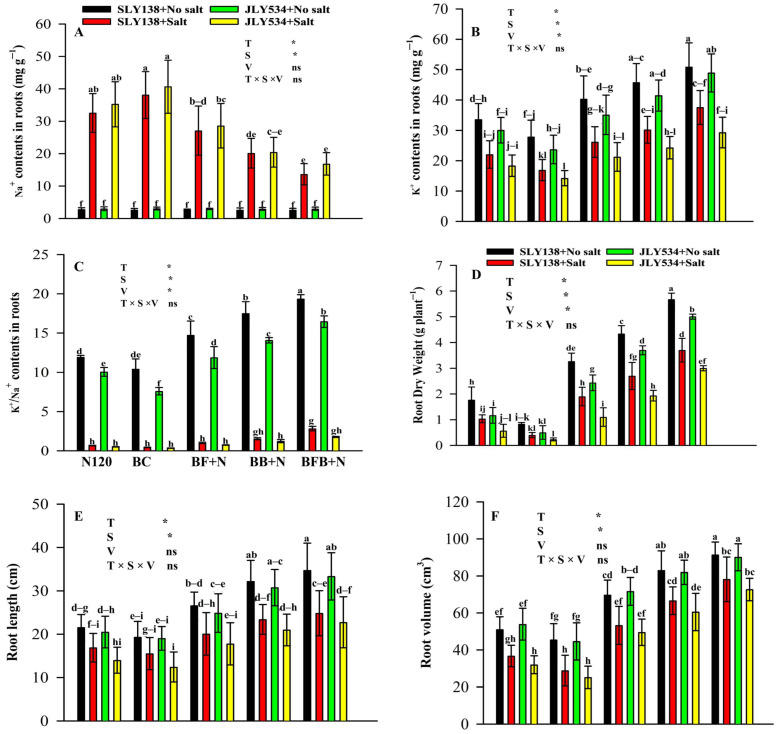
Effect of microbial-inoculated biochar and N fertilizer on Na^+^ and K^+^ of rice plant roots: (**A**) Na^+^ content in roots, (**B**) K^+^ content in roots, (**C**) K^+^/Na^+^, (**D**) root dry weight, (**E**) root length, and (**F**) root volume. The means that have the same letter do not differ substantially at *p* > 0.05 for a parameter. Significant * (*p* ≤ 0.05); non-significant (ns; *p* > 0.05). T (treatment); S (salt); V (variety).

Rice roots exposed to saline water accumulated substantially higher Na^+^ concentrations than those grown under non-saline conditions (Figure 7A). Under saline stress, the BFB+N120 treatment significantly reduced root Na^+^ accumulation compared with N120 and BC treatments alone, showing reductions of 58% and 64% in SLY138 and 52% and 58% in JLY534, respectively. In contrast, root K^+^ content was markedly enhanced under BFB+N120, increasing by 70% and 122% in SLY138 and by 60% and 106% in JLY534 relative to N120 and BC treatments (Figure 7B). Consequently, the pronounced differences in Na^+^ and K^+^ accumulation resulted in a substantially higher K^+^/Na^+^ ratio under BFB+N120, which increased by 318% and 538% in SLY138 and by 236% and 397% in JLY534 compared with BC and N120 treatments, respectively (Figure 7C).

Consistent with these ionic adjustments, the combined application of bacterial–fungal biochar and nitrogen enhanced rice root morphological traits under saline conditions (Figure 7D–F). Specifically, BFB+N120 markedly increased root dry weight, with increases of 258% and 825% in SLY138 and 429.4% and 1185.7% in JLY534 relative to N120 and BC treatments, respectively (Figure 7D). Root length also increased under BFB+N120 by 47.2% and 60.0% in SLY138 and by 62.8% and 83.4% in JLY534 compared with N120 and BC treatments (Figure 7E). Similarly, root volume was significantly enhanced by BFB+N120, increasing by 112.8% and 45.1% in SLY138 and by 126.7% and 188.1% in JLY534 relative to N120 and BC treatments, respectively (Figure 7F).

### 2.7. Pearson Correlation, Cluster Heat-Map and SEM Analysis

**Figure 8:**
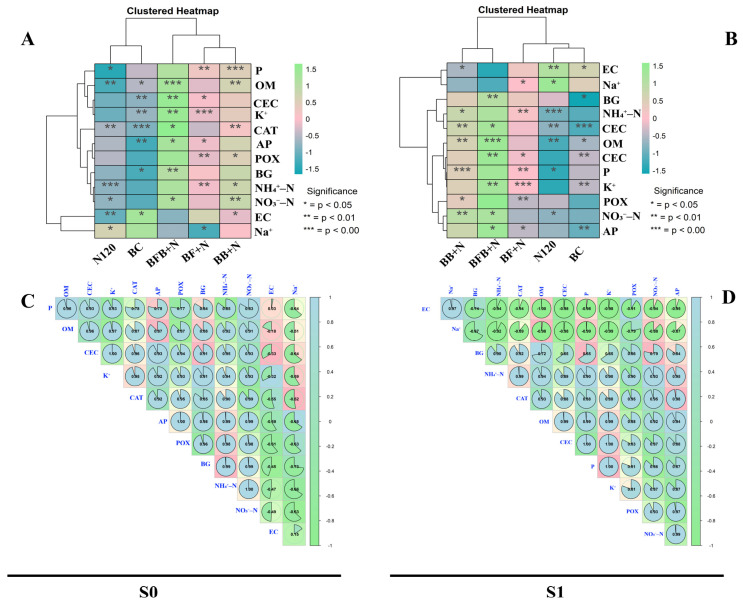
Effect of microbial-inoculated biochar and N on soil properties and correlation analysis between soil parameters. (**A**) Cluster heat-map for S0, (**B**) cluster heat-map for S1, (**C**) correlation analysis for S0, and (**D**) correlation analysis for S1. S0 (no salt); S1 (salt—0.4%).

The colored grid represents the magnitude of the relationships (Figure 8A,B). Warmer colors (red, pink) indicate positive correlations, while cooler colors (blue, green) suggesting negative correlations. The intensity of the color reflects the strength of the relationship. The dendrograms on the left and top indicate how the soil properties and treatments were grouped based on their similarities. This helps identify patterns and potential groupings. The asterisks (*) provide information about the statistical significance of the relationships. More asterisks generally mean a higher level of significance.

The plot shows relationships between various soil properties like pH, organic matter (OM), cation exchange capacity (CEC), and different forms of nitrogen (NH_4_^+^-N, NO_3_^−^-N). The practices include different combinations of nitrogen (N120), simple biochar (BC), and combinations of microbial-inoculated biochar and N (BF+N, BB+N and BFB+N) applications. Some properties, like P, OM, K^+^ and CEC, seem to be consistently positively correlated with BC, BF+N, BB+N and BFB+N. Enzyme activities like AP, POX, BG and CAT also show a positive correlation with BF+N, BB+N and BFB+N. On the other hand, Na^+^ and EC of soil show a negative correlation with these treatments. Other relationships appear more complex, with correlations varying depending on the specific combination of practices. The significance levels provide insights into which relationships are more robust.

Pearson correlation between soil properties showed that Na^+^ and EC of soil are negatively correlated with all other soil properties like OM, P, K^+^, CEC and enzyme activities, while all other soil properties are positively correlated with each other (Figure 8C,D).

The plot suggests that different land management practices can have varying impacts on soil properties. Some practices might be more effective than others in improving certain soil properties. The clustering analysis reveals potential groupings of practices with similar effects on soil.

**Figure 9:**
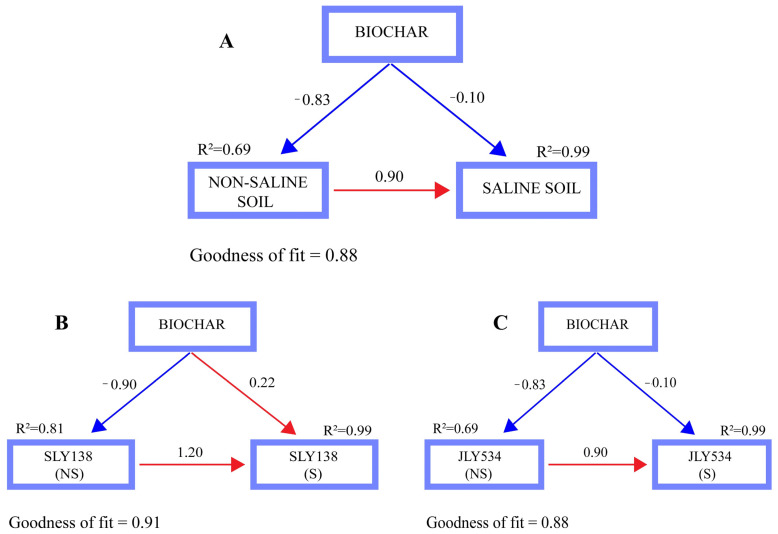
The structural equation model shows the relationship of biochar with soil (**A**), roots of rice cultivar SLY138 with biochar (**B**) and roots of JLY534 with biochar (**C**). Red and blue arrows depict the positive and negative relationships, respectively. Numbers near the pathway arrow indicate the standard path coefficients (r). R^2^ represents the proportion of variance explained for every dependent variable.

Structural equation model (SEM) showed (Figure 9A) that microbial-inoculated biochar with N had a negative effect on soil properties under non-saline (r = −0.83) and saline conditions (r = −0.10). Biochar also had a negative effect (r = −0.90) on the roots of SLY138 under non-saline conditions, while roots of SLY138 were positively influenced (r = 0.22) under saline conditions (Figure 9B). The roots of JLY534 were negatively influenced ((r = −0.83) and (r = −0.10)) under non-saline and saline conditions, respectively (Figure 9C).

## 3. Discussion

The decline in free-living microbial populations can be attributed to the combined effects of Na^+^ and Cl^−^ ion toxicity and abrupt shifts in soil pH following salt and biochar application. This observation aligns with findings from [34], who emphasized the detrimental impact of sudden changes in soil pH, as reported by Wei et al. [35], on microbial community dynamics. The observed initial decline in bacterial and fungal densities (Figure 1F) can be attributed to the physiological stress experienced by microorganisms upon exposure to the saline environment. Upon colonization of an external environment, microorganisms face challenges including abiotic stressors and biotic competition from indigenous microbial communities, which can impede their adaptation to edaphic conditions [36]. However, the subsequent increase in inoculated microbial populations can be ascribed to two primary factors: (1) The controlled release of microorganisms from the biochar matrix, mitigating their immediate exposure to the harsh environmental conditions, as previously reported by Wei et al. [35]. (2) The proliferation of microbial populations within the biochar matrix (BF, BB and BFB) following adaptation to the saline environment, as documented by [15], as evidenced in Figure 1F. These findings indicate that biochar has a positive effect on microbial proliferation. In many studies, it was reported that biochar provides a favorable habitat to protect microbes from predators and unfavorable environmental conditions, and supplies essential nutrients and water, thereby stimulating microbial growth [37,38]. In their study, Zhang et al. [39] reported that microbial decomposition or adsorption of biochar’s insoluble organic and inorganic constituents during microbial proliferation induces alterations in biochar structure, subsequently impacting microbial community dynamics. Similarly, biochar serves as a source of essential mineral nutrients, which, upon gradual release into the soil, enhance soil fertility and consequently stimulate microbial proliferation [39]. Furthermore, biochar possesses the capacity to adsorb and mitigate the bioavailability of salts (Na^+^ and Cl^−^) to microbial populations [40,41]. The observed different survival rates of bacteria and fungi within the biochar matrix indicate that fungal colonization was less pronounced compared to bacterial colonization. This disparity can be attributed to the presence of elevated levels of mineral elements and organic compounds within the biochar, which may exert inhibitory effects on fungal growth, as reported by Lehmann et al. [42]. Furthermore, abrupt shifts in soil pH, as documented by Chen et al. [43], can further exacerbate the challenges faced by fungal populations within the biochar environment. It was reported that within specific ranges of salinity, certain bacterial strains exhibit enhanced growth [44]. Furthermore, bacterial populations generally demonstrate greater salt tolerance compared to fungal counterparts [45]. Fungal sensitivity to salinity stress may be attributed to a substantial reduction in ergosterol content upon salt exposure [46]. Moreover, inherent genetic variations among microbial species likely play a crucial role, either directly or indirectly, in determining their survival and reproductive success within saline environments.

In the present study, the effects of microbial, modified biochar on soil physicochemical properties were investigated to elucidate the mechanisms by which microbial inoculation enhances the ability of biochar to remediate the adverse impacts of saline irrigation on soil. Saline irrigation increased soil electrical conductivity (EC) and sodium (Na^+^) concentration while decreasing available phosphorus (P), cation exchange capacity (CEC), organic matter (OM), potassium (K^+^), ammonium nitrogen (NH_4_^+^-N), and nitrate nitrogen (NO_3_^−^-N). Our findings also align with previous reports, indicating a detrimental effect of saline irrigation water on soil chemical properties [47,48]. Soil EC is a critical indicator of soil salinity, with anions such as Cl^−^ contributing to increased electrical conductivity [49]. The incorporation of Penicillium oxalicum, an alkali-tolerant, acid-producing fungus, into biochar resulted in a reduction in EC [50]. Salinity inhibited organic matter accumulation that was likely attributed to reduced primary productivity and decreased organic matter input quality in highly saline conditions [51]. Moreover, salinity makes OM more susceptible to loss due to its negative impacts on soil aggregate formation [52]. Carbon mineralization was reduced either by the direct adsorption of labile organic compounds or by their stabilization within the soil matrix by biochar [53,54]. According to that, soil degradation results in reduced soil permeability, depletion of essential nutrients (e.g., nitrogen, phosphorus, and organic carbon), and decreases their availability [55].

Conversely, biochar-based microbial amendments combined with nitrogen (N) mitigated these adverse effects by decreasing soil EC and Na^+^ levels, while increasing CEC, OM, P, NH_4_^+^-N, and NO_3_^−^-N in saline soils (Figure 2). The improvement of soil properties was directly proportional to microbial inoculation into biochar. Soil physicochemical properties were improved in the order of BFB+N120 > BB+N120 > BF+N120 > BC. Saline water elevated soil EC, while BFB with N treatment significantly reduced it (Figure 2B). This might be due to the high bio-adsorption capacity of microbial-modified biochar (BF, BB and BFB) and the absence of salt ions (Na^+^ and Cl^−^) within the material, as confirmed by elemental analysis (Figure 1B). Conversely, nitrogen (N) had an insignificant effect on soil EC [56]. Similarly, bacteria and fungi can increase soil organic matter (OM) by decomposing biochar and soil nutrients, as well as by excreting extracellular polysaccharides [57]. In our study, microbial modified biochar (BFB) increased available nutrients (phosphorus, NH_4_^+^-N and NO_3_^−^-N), CEC and OM (Figure 2). The variation in CEC was closely related to biochar and the original soil properties.

Compared to simple biochar, increases in P, NH_4_^+^-N and NO_3_^−^-N, CEC and OM may be due to the inoculation of microbes into biochar (BFB). The heterogeneous response of microorganisms results in shifts in microbial community composition, ultimately influencing soil biogeochemical cycling and ecosystem function [55], as microorganisms drive key biogeochemical processes through the production of extracellular and intracellular enzymes, which serve as vital indicators of soil quality due to their roles in microbial activity and nutrient cycling. Biochar amendments can enhance enzyme activities, thereby improving degraded soil biological properties [58,59,60]. Consistent with these findings, our study showed that the treatment exhibiting the greatest improvement in soil properties also demonstrated enhanced enzyme activities (Figure 6). Moreover, Zheng et al. [61] also reported that electrostatic forces help the biochar to adsorb nutrients like phosphorus, NH_4_^+^-N and NO_3_^−^-N, while microbial activity enhances nutrient concentration through soil mineralization processes, indicating the potential for microbial nitrogen fixation in wastewater [62]. Similarly, biochar, as a significant edaphic amendment, influences soil physiochemical properties (e.g., pH, nutrient availability, and water-holding capacity) and elicits a variable response within the soil microbial community [36]. Biochar reduces the immediate availability of phosphorus due to its adsorption capacity, while microbes can uptake and store it, gradually enhancing its availability upon decomposition [63].

Exchangeable soil Na^+^ concentrations were higher under saline compared to non-saline conditions, while microbial-modified biochar (BF, BB, and BFB) showed N-reduced soil Na^+^ under saline conditions (Figure 2H). Soil-exchangeable K^+^ concentration increased across all treatments but varied significantly among them (Figure 2G). Biochar may reduce soil Na^+^ by accelerating its exchange through improved soil physical properties, as suggested by Jin et al. [64]). In our study, inoculated microbes (BF, BB, and BFB) enhanced soil physicochemical properties more effectively than BC (Figure 2). Moreover, Dahlawi et al. [65] suggested that biochar-released cations like Ca^2+^ and Mg^2+^ displace Na^+^ from cation exchange sites and the soil solution. Characterization of our biochar shows that these bivalent cations are high in microbial-modified biochar like BF, BB and BFB compared to BC (Figure 1B). Inoculated and activated native microbes might enhance biochar’s ability to reduce soil Na^+^ through bioaccumulation, as halophilic microbes can absorb and potentially export sodium ions under elevated salinity [49]. Microbial inoculant biochar (BFB) applications increased microorganisms possessing Na^+^/H^+^, Na^+^/Ca^2+^, and Na^+^/K^+^ transport proteins, aligning with the findings from [45]. Additionally, it was also reported that nitrogen (N) had an insignificant effect on soil EC [56]. The influence of biochar on soil ion concentrations exhibits complexity and depends upon experimental parameters [11]. For example, it was reported that the application of biochar to saline soils resulted in the adsorption of Na^+^ and the concurrent release of K^+^, Ca^2+^, and Mg^2+^ ions, particularly when the biochar was acidified [66].

Potential mechanisms of microbial biochar in enhancing soil properties include the following: (1) The adsorption of extracellular polymeric substances (EPSs) onto the biochar surface, forming a protective matrix that mitigates external environmental stressors and provides an auxiliary energy source for microorganisms, thereby stimulating microbial proliferation and metabolic activity. This process facilitates soil organic matter transformation and enhances soil structural integrity [67]. (2) Microorganisms release nutrients, including carbohydrates, proteins, and peptides, via extracellular polymeric substances (EPSs). These substances can adsorb onto the biochar surface or engage in chemical interactions, forming a biochar–microbial complex. This complex facilitates nutrient exchange and transfer among microorganisms, thereby enhancing nutrient bioavailability within the soil matrix [36]. (3) Biochar modulates microbial metabolic activities and enhances soil fertility and biological activity by adsorbing microbial signaling molecules (e.g., CO_2_, flavonoids, terpenoids), thereby influencing intercellular communication and extracellular polymeric substance (EPS) secretion [68]. The combined application of biochar, microbial inoculation, and nitrogen fertilizer showed a combined effect under salinity stress, as the improvement in soil organic matter, nutrient availability, and microbial activity was significantly greater than that observed under individual treatments.

Following an initial increase from the pre-experimental soil pH at day 5, all treatments except BC exhibited a decreasing trend until saline water irrigation at day 20 (Figure 3). Previously, many studies reported the same fluctuations in soil pH within the incubation from biochar and microbial-modified biochar [69]. They proposed that the rapid initial soil pH increase resulted from alkaline substance dissolution (e.g., inorganic carbonate) within the biochar, followed by a minimal pH alteration after the depletion of these readily released alkaline compounds. Combined bacterial–fungal-loaded biochar with N treatment induced a more pronounced pH decrease compared to simple biochar and N120 treatment. Specifically, the BFB+N treatment significantly decreased soil pH (*p* < 0.05) following the 20-day incubation (Figure 3). It was reported by Du et al. [70] that the minor reduction in soil pH might be attributed to proton (H^+^) release during the soil nitrification process, as nitrification is an acidifying process and causes a reduction in soil pH [71,72]. Current study findings suggested that the effect of microbial-modified biochar and N can effectively mediate the pH of soil irrigated with saline water during the whole incubation period. Saline water flooding triggers reduction reactions that consume H^+^ ions, leading to increased soil pH [73]. Bacterial and fungal secretions may contain low-molecular-weight organic acids [22], potentially causing a greater soil pH reduction in BFB+N treatments (Figure 3).

Elevated salinity adversely impacts soil microbial population size, activity, and diversity [74]. In another study, it was also reported that soil degradation results in reduced soil permeability and compromised microbial activity [55]. Conversely, microbial inoculation on biochar with N enhanced bacterial and fungal abundance in saline paddy field rhizospheres compared to biochar treatment (Figure 4). According to Li et al. [75], nitrogen substrates and other physicochemical properties affected soil microbial diversity and community composition. Some bacterial phyla, like Proteobacteria, Actinobacteriota, Chloroflexi, and Firmicutes, were identified as the dominant bacterial phyla across all soil samples (Figure 4A). Consistent with the findings by Huang et al. [76] and Gu et al. [77], dominant bacterial phyla including Proteobacteria, Actinobacteriota, Chloroflexi, and Firmicutes were identified across all soil samples. Moreover, according to Hobbie et al. [78], the application of N could reduce the N requirement for microbial growth and decreased production of enzymes involved in N-acquisition. Many of these microbes improved the physicochemical properties of degraded soil, as Proteobacteria, the predominant salt-tolerant bacterial phylum in saline soils, significantly contribute to soil nitrogen cycling [23,79,80]. Moreover, Samaddar et al. [81] and Sun et al. [82] reported the sensitivity of actinobacteriota community to N, highlighting their crucial role in soil nitrogen cycling. According to Cai et al. [67], Chloroflexi are halotolerant and are found in more abundance in eutrophic environments. Notably, Proteobacteria, Actinobacteriota, Chloroflexi and Firmicutes abundance did not significantly differ among treatments, possibly due to saline water influence (Figure 4B). All these bacteria were reported in soil with nutrient-rich conditions [83,84]. Moreover, according to Chen et al. [84], Acidobacteria, known for their oligotrophic nature, thrive in nutrient-limited environments, exhibiting increased relative abundance in resource-scarce soil ecosystems. Our study revealed that microbial-modified biochar (BF, BB, and BFB) with N significantly reduced Acidobacteria-relative abundance compared to BC treatment, indicating improved soil nutrition through these modified biochars and N (Figure 4B). Desulfobacterota and Bacteroidota, identified as pollution-removing bacteria [49], are salt-resistant and can remediate saline soil [85,86]. Bacteroidota proliferation is enhanced by nutrient-rich biochar- and N-derived organic matter [86]. According to Kruczyńska et al. [87], the abundance of bacteroidota serve as a key indicator of soil biological remediation. Moreover, Zhang et al. [49] reported that bacteroidota growth indicates an enhanced capacity to digest complex organic compounds, supporting aquaculture wastewater purification. Similarly, BFB+N treatment has relatively more abundance of Ascomycota and basisdiomycota fungi as compared to BC and N120 treatments alone (Figure 4C). Many researchers reported that Basidiomycota and Ascomycota are saprotrophic fungi, which are more able to degrade lignocellulose organic matter [88,89,90]. Co-application of microbial-modified biochar and N increased soil bacterial taxa abundance, promoting symbiotic relationships (Figure 4) and enhancing soil nutrient cycling [91]. Our study also revealed a combined effect of microbial biochar addition and N on the relative abundance of Mortierellomycota (Figure 4C). According to Johnson et al. [92], Mortierellomycota are beneficial fungi and improve nutrient conditions in contaminated soil, and they also help the plants by enhancing their resistance during the symbiosis process.

Biochar can enhance biological properties of soil in different ways. For example, biochar functions as a complex carbon and nutrient reservoir, supporting microbial growth and enzymatic activity [17,59]. Similarly, Domingues et al. [93] reported that biochar promotes microbial activity by enhancing available nitrogen (NO_3_^−^-N). Applying microbial-modified biochar and nitrogen to high-salinity environments enhances soil microbial community richness and stability. The combined effect of microbial biochar and nitrogen enhances the structural integrity, functional capacity, and stability of soil microbial networks more effectively than when either is applied alone.

Similar to microbial abundance, salinity also affects soil enzyme activities (Figure 6). Previous studies have widely reported that salt stress negatively affects soil enzyme activity [94,95]. However, the addition of biochar may enhance soil properties and resultantly improve soil enzyme activities [96]. In the present research, BFB+N significantly enhanced the activities of acid phosphatase (AP), catalase (CAT), polyphenol oxidase (POX) and β-D-Glucosidase (BG) in saline soil (Figure 6). Many other studies have also reported that different types and concentrations of biochar can activate soil enzymes [97,98]. According to Song et al. [99], activities of acid phosphatase and CAT were increased due to biochar. Similarly, microbes (bacteria and fungi) can also produce enzymes such as polyphenol oxidase [100], and fungi are the main contributors to oxidase production [101,102]. Interestingly, microbial enzyme synthesis and activity are strongly dependent on nutrient availability, with microbes prioritizing resource allocation towards enzyme production in response to nutrient limitation [17]. In their studies, Qi et al. [103] and Shang et al. [104] reported that, in saline soils, frequently exhibiting nutrient limitations, microbial communities typically exhibit increased enzyme production to facilitate the mineralization and cycling of scarce nutrients, thus improving their bioavailability for plant assimilation. Also, N has positive effects on soil enzyme activities [105]. Soil quality emerges from the dynamic interactions among soil physical and chemical properties and microbial communities, whereby improvements in organic matter, nutrient availability, and cation exchange capacity directly enhance microbial abundance and enzymatic activity, ultimately regulating soil functionality and crop productivity under saline conditions [106]. Improvements in soil physicochemical properties induced by biochar amendments—particularly increases in soil organic matter, nutrient availability, and pH regulation—directly shape microbial community structure and interactions by enhancing habitat quality and substrate availability, thereby promoting the proliferation of functionally important bacterial taxa involved in nutrient cycling and stress mitigation under saline conditions [107].

Plant roots possess a polarized anatomy, with the outer layers (epidermal and cortical cells) responsible as the first organ to uptake K^+^, Na^+^ and other nutrient ions [108,109]. Our study showed that Na^+^ was increased and K^+^ content was decreased in roots under saline conditions compared to non-saline conditions for both cultivars (Figure 7). However, microbial-inoculated biochar and N act oppositely to salinity. Similarly, it was reported that biochar application decreased Na^+^ content in roots due to its adsorption capacity either by reducing Na^+^ uptake, excluding Na^+^, or both [110]. In another study, Zhu et al. [111] also reported that the application of biochar in saline soil decreased Na^+^ content and increased k^+^ in roots. Basically, biochar application improved cation exchange capacity (CEC) of soil [112,113]. Moreover, Akhtar et al. [110] reported that biochar application can enhance K^+^ concentrations and reduce Na^+^ uptake, in addition to its strong capacity for Na^+^ adsorption. Additionally, high K^+^ concentrations in roots might be attributed to the presence of locally available nutrients, particularly exchangeable K^+^, in the soil [113].

In contrast, the antagonistic effect of N with Na^+^ and the cordial effect with K^+^ in roots was also reported [114,115,116]. Moreover, a positive relation between N and salinity was reported when applied to the maize crop [117]. Similarly, Javed et al. [9] also reported that increasing N fertilizer applications can mitigate Na^+^ uptake in plants due to the antagonistic effect between Na^+^ and K^+^ ions, favoring K^+^ ions. The reason behind this positive response of N application might be due to increased root growth, which ultimately improves nutrient uptake and mitigates the detrimental effects of Na^+^ toxicity [118].

Salt stress has detrimental effects on root morphological characteristics and anatomical parameters [119]. Similar to these findings, our study reports that salt stress has a negative effect on root biomass, root length and root volume (Figure 7D–F). The BFB+N treatment also greatly improved the structure of root tips for SLY138 under salt stress (Figure 7D). Previously, in many studies, ultrastructural changes of roots were improved by biochar and N under salt stress [120]. Moreover, microbial inoculants ameliorate salt stress in plants via an increased nutrient uptake, induced antioxidative defense system, modulation of the level of plant hormones, and reduction of the ethylene level by producing 1-aminocyclopropane-1-carboxylate-deaminase in the plant rhizosphere [15]. Similarly, Zhang et al. [121] reported that N is a major nutrient element for plant growth and its application to soil improves roots attributes. Nitrogen, predominantly absorbed by plant roots as ammonium (NH_4_^+^-N) and nitrate (NO_3_^−^-N), is a critical nutrient essential for plant growth [122]. On the other hand, Li et al. [123] reported that excessive nitrogen (N) application decreased the total fine root surface area but increased the thickness of individual fine roots, potentially leading to an increase in total root biomass. Our study suggests that nitrogen fertilizer acts as a supportive element for biochar and microbes under saline conditions, enhancing their ability to promote root growth.

## 4. Materials and Methods

### 4.1. Microbial Biochar Preparation and Characterization

The *Mycobacterium* sp. and *Penicillium* sp. were used to prepare bacterial-, fungal- and bacterial–fungal-loaded biochar (BB, BF and BFB, respectively). The inoculants (*Mycobacterium* sp.-191574 and *Penicillium* sp.-353380) were purchased from “BeNa Culture Collection” Beijing, China (BNCC) and further multiplied in our Lab. Lowenstein–Jensen medium and potato dextrose agar (PDA) medium were obtained from Merck, Darmstadt, Germany, and prepared following the manufacturer’s guidelines. Before inoculations, microbial inoculants (bacteria and fungi) were grown in “Lowenstein–Jensen medium” and “PDA media”, Merck, Darmstadt, Germany, respectively, in a shaking incubator at 120 rpm and 30 °C for 72 h. These microbial modified biochars (BB, BF and BFB) were prepared by physical adsorption. Microbial modified biochars were prepared by 1:10 biochar/microbial suspension containing actively growing cultures. The inoculum was applied by soaking the biochar in the suspension under gentle agitation to allow microorganisms to penetrate the pore structure and placed in a shaking incubator at 120 rpm and 30 °C for 24 h to facilitate microbial attachment and colonization. After the inoculation of bacteria and fungi onto the simple biochar (BC), the composite materials (BB, BF and BFB) were collected and freeze-dried. The surface morphology of biochar (BC) and bacterial–fungal-loaded biochar (BFB) were analyzed using a scanning electron microscope (German, Zeiss Sigma 300, Carl Zeiss AG, Oberkochen, Germany), while, XRD, EDS and FTIR of BFB were analyzed using (JP Rigaku Smart Lab SE, Rigaku Corporation, Tokyo, Japan), (OxfordX-MAX, Oxford Instruments, Abingdon, UK) and (Thermo Fisher Scientific, Madison, WI, USA), respectively.

### 4.2. Aliveness of Free and Biochar-Inoculated Microbes Under Salt Stress Conditions

When comparing the survival rates of free-living and biochar-immobilized microbial populations within saline soils, it is critical to establish equivalent initial microbial concentrations within the soil matrix for both groups. The living cell numbers of bacteria, fungi, fungal-immobilized biochar (BF), bacterial-immobilized biochar (BB) and bacterial–fungal-inoculated biochar (BFB) were measured as described by Luo et al. [124]. To determine microbial viability, 1 mL of each bacterial and fungal culture, or 1 g of the microbial-loaded material, was aseptically transferred to 50 mL of sterile water and homogenized using a vortex mixer. Serial dilutions of the resulting suspension were subsequently performed. Aliquots of 100 μL from each dilution were then plated onto LB agar plates. The inoculated plates were incubated at 30 °C for 24 h, followed by enumeration of colony-forming units (CFUs). To ensure equivalent viable microbial inoculant across treatments, 1 mL of Mycobacterium spp. (2 × 10^10^ CFU mL^−1^) and 1 mL of Penicillium spp. (2 × 10^10^ CFU mL^−1^) were added to 10 g of sterilized soil, respectively. Similarly, 0.48 g of fungal-loaded biochar (BF, 3.9 × 10^10^ CFU g^−1^), 0.3 g of bacterial-loaded biochar (BB, 4.5 × 10^10^ CFU g^−1^) and 0.26 g of bacterial–fungal-loaded biochar (BFB, 4.9 × 1010 CFU g^−1^) were incorporated into 10 g of sterilized soil. All treatments were incubated in the dark at 30 °C. Saline stress was imposed on the soil by irrigating with a 0.4% NaCl solution. Soil moisture content was maintained at 60% of field capacity through regular additions of distilled water. To monitor temporal changes in microbial populations, soil samples were collected at 0, 10, 20, 30, and 40 days. The number of viable microorganisms (CFU g^−1^) in each sample was quantified using the previously described plate count method.

### 4.3. Experimental Setup, K^+^/Na^+^ in Rice Roots and Root Morphology

A 70-day study was conducted in a glass house at the research area of Hainan University, China, under a completely randomized block design with three replications in plastic pots with (15 cm × 22.5 cm × 20.5 cm) and with each pot filled with 5 kg soil. The soil was collected from a typical forest (C. nucifera) in Lingao County located in the northwestern part of Hainan Island, China. The physicochemical properties of this soil were determined according to the methods described by Xue et al. [125]. The physicochemical properties of pre-experimental soil used in this study are presented in Table 2. The rice straw-derived biochar was used for microbial inoculation and was sieved to 0.2 mm. Its nutrient contents were analyzed by an elemental analyzer and listed in Table 3. Following this, various combinations of biochar and nitrogen with (No salt and salt) were introduced to the soil, either individually or in combination. Detailed information of the applied treatments is presented in Table 1. Seeds were surface-sterilized and germinated prior to sowing, and after emergence, six uniform seedlings per pot were maintained for subsequent growth. Plant growth was monitored throughout the experiment, and growth parameters including plant height, root length, biomass accumulation, and root morphology were measured at harvest after a defined growth period. Further, on the basis of the morphological attributes of rice plants, the best-performing treatments were selected for further analysis of soil and rice roots and were represented (Table 4). Two rice cultivars were used in this experiment: (1) the salt-resistant Shuang Liang You 138 (SLY138) and (2) the salt-sensitive Jing Liang You 534 (JLY534). These cultivars were subjected to two different levels of NaCl salinity: 0% (control-S0) and 0.4% (S1). Biochars (BC, BF, BB and BFB) were mixed 30 g pot^−1^ in soil before pot filling, and nitrogen (N) fertilizer (Table 1) was applied in three splits (sowing, seedling stage, and 40 DAS) respective to the treatment applied. Salt was applied after 20 days of rice sowing until 0.4% salt level was maintained in pots irrigated with NaCl salt solution respective to the salt-applied pots, while control pots were irrigated with tap water.

To measure morphological attributes, roots were washed to remove dust and scanned by a root scanner (Epson Perfection V370 Photo, Seiko Epson Corporation, Nagano, Japan) and then analyzed by a root analyzer. Total K^+^ and Na^+^ content in roots were determined with the flame photometer method, according to [126].

### 4.4. Physicochemical and Biological Properties of Post-Harvested Soil

After the experiment, soil samples were collected from the rhizosphere of each replicate for both rice varieties. These fresh samples were then thoroughly mixed to create a single composite sample for each variety. The composite samples were dried and sieved through a 2 mm mesh to prepare them for further analysis.

To analyze the effects of modified biochar and N on microbial communities, rhizosphere soil samples were collected and subjected to DNA extraction using a TGuide S96 Magnetic Sol/Stool DNA Kit, Tiangen Biotech Co., Ltd., Beijing, China. DNA quality and quantity were assessed using gel electrophoresis and Nano Drop spectrophotometry, Bio-Rad Laboratories, Hercules, CA, USA. The V3–V4 hypervariable region of the bacterial 16S rRNA gene was amplified using Illumina-indexed primers 338F (5′-ACTCCTACGGGAGGCAGCA3′) and 806R (5′-GGACTACHVGGGTWTCTAAT-3′). PCR products were purified, quantified, and sequenced on an Illumina NovaSeq6000 platform, (Illumina, Inc., San Diego, CA, USA) using paired-end sequencing technology.

Soil enzyme activities, including acid phosphatase (AP) and catalase (CAT), were determined using a method described by James et al. [127]. β-D-Glucosidase (BG) and polyphenol oxidase (POX) activities were determined according to the methods described by [128] and [129], respectively.

Moreover, soil physicochemical properties were determined after the experiment using standard analytical methods. Organic matter content was determined using the Walkley--Black method [130,131]. Soil pH and electrical conductivity (EC) were measured in 1:2 and 1:5 soil/water suspensions, respectively, using pH and EC meters. Soil CEC was assessed by extraction with 1.0 M ammonium acetate followed by titration with 50 mM hydrochloric acid. Flame photometry was employed to quantify soluble and exchangeable sodium (Na^+^) and potassium (K^+^) content. The available nitrogen content including ammonium nitrogen, and nitrate nitrogen was determined using the indophenol blue method [132]. Available phosphorus (P) was extracted from the soil samples using a sodium bicarbonate (NaHCO_3_) solution and subsequently quantified spectrophotometrically using a Clever Chem Anna analyzer at a wavelength of 700 nm [133].

### 4.5. Statistical Analysis

Statistical analyses were performed using SPSS version 8.1. Prior to analysis, data were inspected for normality and homogeneity of variances. While most variables approximately met ANOVA assumptions, it is acknowledged that germination percentages and leaf numbers represent proportion or count data, which may deviate from normality; however, the approximation was considered reasonable for the applied analyses. Interaction effects among biochar, microbial inoculation, nitrogen fertilization, and salinity were evaluated using factorial ANOVA. The combined treatment responses were compared with those of individual treatments to assess potential interaction effects, and improvements observed under the combined application were interpreted as indicative of positive interactions among the applied treatments.

Treatment effects, excluding microbial abundance, were presented as means ± standard deviation (*n* = 3). A two-way or three-way analysis of variance (Table 1) was used to evaluate the effects of biochar + nitrogen, variety (plants related attributes) and salinity treatments, followed by least significant difference (LSD) tests at *p* < 0.05. Factorial interactions were examined; when interactions were not significant, main effects were reported separately for clarity and precision.

Pearson correlation and cluster heat-map analyses were performed using R software version 4.4.1. Correlation and heat-map plots were constructed with the packages corrplot, extrafont, viridis, dplyr, stats, ggplot2, metan, and RColorBrewer.

RStudio (version 4.3.1) was used for Structural equation model (SEM) analysis, and the partial least squares path modeling (PLS-PM) was performed using the plspm package (version 0.5.1) to evaluate the relationships among soil physicochemical properties, microbial activity, plant physiological traits, and salinity stress.

## 5. Conclusions

This study demonstrates that the combined application of bacterial–fungal biochar with nitrogen represents an effective strategy for mitigating soil salinity stress. Rather than acting solely as a soil amendment, microbial-inoculated biochar functioned as a multifunctional regulator by improving root system architecture, enhancing soil nutrient availability, reducing sodium accumulation, and stimulating microbial abundance and enzymatic activity. The persistence of dominant microbial taxa such as Proteobacteria and Ascomycota across treatments further indicates stable microbial establishment and functional resilience in saline soils. Collectively, these findings highlight the combined interactions among biochar, microorganisms, and nitrogen in restoring soil biological functionality and plant performance under saline conditions. Given its cost-effectiveness and practical applicability, microbial-inoculated biochar has a strong potential as a sustainable tool for improving productivity and ecological stability in saline and saline–alkaline soils.

## Figures and Tables

**Table 1:** Treatment plan.

Treatment	Salt Levels	Treatment Name	Application Rates
T1	0%	N60	Nitrogen (60 kg ha^−1^)
	0.40%	N60	Nitrogen (60 kg ha^−1^)
T2	0%	N120	Nitrogen (120 kg ha^−1^)
	0.40%	N120	Nitrogen (120 kg ha^−1^)
T3	0%	BC	Simple Rice Straw Biochar (20 t/ha)
	0.40%	BC	Simple Rice Straw Biochar (20 t/ha)
T4	0%	BF	Fungal-Inoculated Biochar (20 t/ha)
	0.40%	BF	Fungal-Inoculated Biochar (20 t/ha)
T5	0%	BB	Bacterial-Inoculated Biochar (20 t/ha)
	0.40%	BB	Bacterial-Inoculated Biochar (20 t/ha)
T6	0%	BC+N60	Simple Rice Straw Biochar (20 t/ha) + Nitrogen (60 kg ha^−1^)
	0.40%	BC+N60	Simple Rice Straw Biochar (20 t/ha) + Nitrogen (60 kg ha^−1^)
T7	0%	BC+N120	Simple Rice Straw Biochar (20 t/ha) + Nitrogen (120 kg ha^−1^)
	0.40%	BC+N120	Simple Rice Straw Biochar (20 t/ha) + Nitrogen (120 kg ha^−1^)
T8	0%	BF+N60	Fungal-Inoculated Biochar (20 t/ha) + Nitrogen (60 kg ha^−1^)
	0.40%	BF+N60	Fungal-Inoculated Biochar (20 t/ha) + Nitrogen (60 kg ha^−1^)
T9	0%	BF+N120	Fungal-Inoculated Biochar (20 t/ha) + Nitrogen (120 kg ha^−1^)
	0.40%	BF+N120	Fungal-Inoculated Biochar (20 t/ha) + Nitrogen (120 kg ha^−1^)
T10	0%	BB+N60	Bacterial-Inoculated Biochar (20 t/ha) + Nitrogen (60 kg ha^−1^)
	0.40%	BB+N60	Bacterial-Inoculated Biochar (20 t/ha) + Nitrogen (60 kg ha^−1^)
T11	0%	BB+N120	Bacterial-Inoculated Biochar (20 t/ha) + Nitrogen (120 kg ha^−1^)
	0.40%	BB+N120	Bacterial-Inoculated Biochar (20 t/ha) + Nitrogen @ 120 kg ha^−1^
T12	0%	BFB+N60	Bacterial–Fungal-Inoculated Biochar (20 t/ha) + Nitrogen (60 kg ha^−1^)
	0.40%	BFB+N60	Bacterial–Fungal-Inoculated Biochar (20 t/ha) + Nitrogen (60 kg ha^−1^)
T13	0%	BFB+N120	Bacterial–Fungal-Inoculated Biochar (20 t/ha) + Nitrogen (120 kg ha^−1^)
	0.40%	BFB+N120	Bacterial–Fungal-Inoculated Biochar (20 t/ha) + Nitrogen (120 kg ha^−1^)

**Table 2:** Physicochemical properties of pre-experimental soil.

	Total Nutrient Content (g/kg)							Available Nutrient Content (mg/kg)			
pH	OM	TN	TP	TK	Moisture Content %	CEC cmol (+)/kg	EC (µs/cm)	AP	AK	Nitrate (N)	Ammonium (N)
6.51	1.05	0.13	0.21	0.65	2.57	2.14	267	4.49	27.35	0.95	1.87

**Table 3:** pH and nutrient status of biochar (BC).

pH	9
CEC cmol (+) kg^−1^	140
Total carbon	80
Nitrogen	2.45
Oxygen	1
Phosphorus	0.09
Potassium	0.14

**Table 4:** Selected treatments for analysis.

Treatment	Salt Levels	Treatment Name	Application Rates
T2	0%	N120	Nitrogen (120 kg ha^−1^)
	0.40%	N120	Nitrogen (120 kg ha^−1^)
T3	0%	BC	Simple Rice Straw Biochar (20 t/ha)
	0.40%	BC	Simple Rice Straw Biochar (20 t/ha)
T9	0%	BF+N120	Fungal-Inoculated Biochar (20 t/ha) + Nitrogen (120 kg ha^−1^)
	0.40%	BB+N120	Bacterial-Inoculated Biochar (20 t/ha) + Nitrogen (120 kg ha^−1^)
T11	0%	BB+N120	Bacterial-Inoculated Biochar (20 t/ha) + Nitrogen (120 kg ha^−1^)
	0.40%	BF+N120	Fungal-Inoculated Biochar (20 t/ha) + Nitrogen (120 kg ha^−1^)
T13	0%	BFB+N120	Bacterial–Fungal-Inoculated Biochar (20 t/ha) + Nitrogen (120 kg ha^−1^)
	0.40%	BFB+N120	Bacterial–Fungal-Inoculated Biochar (20 t/ha) + Nitrogen (120 kg ha^−1^)

## Data Availability

Data will be made available on request.
